# Biogeographical patterns in soil bacterial communities across the Arctic region

**DOI:** 10.1093/femsec/fiz128

**Published:** 2019-08-20

**Authors:** Lucie A Malard, Muhammad Z Anwar, Carsten S Jacobsen, David A Pearce

**Affiliations:** 1 Faculty of Health and Life Sciences, Northumbria University, Newcastle-upon-Tyne NE1 8ST, UK; 2 Department of Environmental Sciences, Aarhus University, 4000 Roskilde, Denmark; 3 British Antarctic Survey, High Cross Madingley Road, Cambridge CB3 0ET, United Kingdom

**Keywords:** 16S rRNA, Arctic soil, microbial diversity, indicator species, core microbiome, biogeography

## Abstract

The considerable microbial diversity of soils and key role in biogeochemical cycling have led to growing interest in their global distribution and the impact that environmental change might have at the regional level. In the broadest study of Arctic soil bacterial communities to date, we used high-throughput DNA sequencing to investigate the bacterial diversity from 200 independent Arctic soil samples from 43 sites. We quantified the impact of spatial and environmental factors on bacterial community structure using variation partitioning analysis, illustrating a nonrandom distribution across the region. pH was confirmed as the key environmental driver structuring Arctic soil bacterial communities, while total organic carbon (TOC), moisture and conductivity were shown to have little effect. Specialist taxa were more abundant in acidic and alkaline soils while generalist taxa were more abundant in acidoneutral soils. Of the 48 147 bacterial taxa, a core microbiome composed of only 13 taxa that were ubiquitously distributed and present within 95% of samples was identified, illustrating the high potential for endemism in the region. Overall, our results demonstrate the importance of spatial and edaphic factors on the structure of Arctic soil bacterial communities.

## Teaser

Arctic soil bacterial communities are influenced by spatial and edaphic factors and exhibit biogeographical distribution.

## INTRODUCTION

Frozen soils in the Arctic region store over 1500 Pg of carbon (Koven *et al*. 2011; Mackelprang *et al*. 2011) and as Arctic warming is exacerbated and permafrost thaw accelerates, the depth of the active layer is increasing. As previously frozen carbon becomes available, it is expected that microbial activity will increase, which may lead to increased atmospheric release rates of climate active gases such as carbon dioxide (CO_2_), methane (CH_4_) and nitrous oxide (N_2_O) (Ma *et al*. 2007; Mackelprang *et al*. 2011). Carbon-climate feedback studies of permafrost affected regions use temperature, soil moisture and precipitation as the main drivers of decomposition rates (Koven *et al*. 2011; Schuur *et al*. 2015). While models are useful to gain a global understanding of the impact of climate change on permafrost thaw and greenhouse gas release, the accuracy of results obtained is highly variable when compared with data collected in the field or laboratory (Schuur *et al*. 2015) due to empirical and modelling uncertainties which still need to be addressed (Bradford *et al*. 2016).

As microorganisms drive biogeochemical cycling and participate in the uptake and release of CO_2_, CH_4_ and N_2_O, microbial data should be incorporated in climate models. Current models use soil properties to model changes in fluxes, without considering microbial communities and the changes in community composition induced by climate change (Bardgett, Freeman and Ostle 2008; Nazaries *et al*. 2013). Adding microbial information into models will improve their predictions; however, detailed microbial data is still required, with a focus on the microbial community, diversity, function, stability and long-term changes in these communities (Graham *et al*. 2012; Nazaries *et al*. 2013). While global surveys of microbial diversity have already been conducted (Tedersoo *et al*. 2014; Delgado-Baquerizo *et al*. 2018), the number of Arctic samples is restricted (Malard and Pearce 2018) and therefore, microbial data is still lacking for most permafrost-affected regions.

To accurately model spatial distribution patterns of microbes and incorporate microbial data from permafrost-affected regions in climate models in the near future, the first step is to produce a baseline database of microbial diversity with respect to biogeographical distribution. Biogeography is the study of biodiversity across space and time; it gives insights into ecological mechanisms such as speciation, extinction, dispersal and species interactions (Martiny *et al*. 2006; Fierer 2008), improving our understanding of community assembly. Theoretically, distant and isolated habitats are expected to present high endemicity as a consequence of intrinsic dispersal limitations and environmental filtering (Mittelbach and Schemske 2015; Kleinteich *et al*. 2017; Bahram *et al*. 2018). Thus, isolated, pristine ecosystems with limited human presence, such as the Arctic region, should harbor endemic communities. However, microbial communities may be less constrained by geographical barriers and thus, have long been considered ubiquitous (Finlay 2002; O'Malley 2007). Yet, recent studies have uncovered patterns of microbial biogeography on global scales (Fierer and Jackson 2006; Lauber *et al*. 2009; Tedersoo *et al*. 2014; Henschel, Anwar and Manohar 2015; Bahram *et al*. 2018; Delgado-Baquerizo *et al*. 2018). They demonstrated that microbial distribution is nonrandom, generally influenced by biotic and abiotic factors such as plant cover, soil pH, C/N ratio or precipitation, and that communities vary from one region to another. However, whether these patterns apply to Arctic microbial communities is still subject to debate as these studies tend to have a low number of Arctic samples and Arctic studies have generally focused on small-scale patterns in restricted areas, as shown by Metcalfe *et al*. (2018) highlighting the focuses on Abisko, Sweden and Toolik lake, Alaska. Large-scale studies are therefore required to improve our understanding not only of the spatial distribution of microorganisms but also on the processes of community assembly. Producing a baseline database of diversity in the region will enable the accurate modelling of spatial distribution, linking with patterns of functional processes to evaluate the potential consequences of environmental change on ecosystem properties.

In this study, we conducted a Pan-Arctic survey of bacterial communities in Arctic soils to provide a baseline of Arctic bacterial diversity. We addressed the following questions: (i) Are bacterial taxa ubiquitously distributed or are there biogeographical patterns? (ii) What are the key factors influencing bacterial community structure? (iii) Are there taxa closely associated with the key edaphic properties and what can we learn from them? (iv) If bacterial taxa are not ubiquitous, is there a core microbiome?

## METHODS

### Sample collection

**Figure 1.**
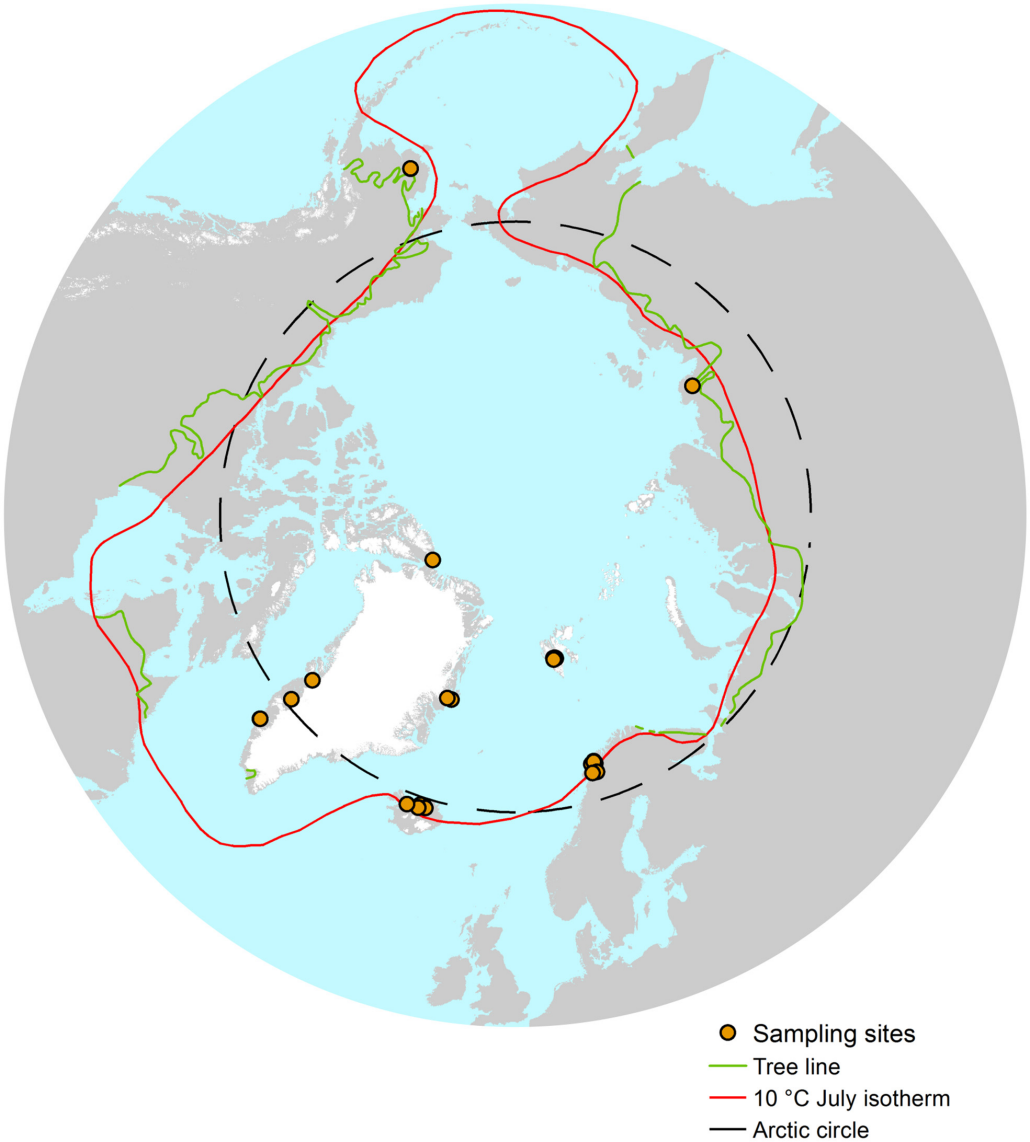
Map of sampling sites within the Arctic Region, as characterized by the Arctic circle (black line), the 10°C isotherm (red) and the tree line boundary (green). 200 individual soil samples at 43 different sites were collected for this study, all within at least one of the definitions of the Arctic region.

Soil samples were collected across the Arctic region between April 2017 and September 2017, and the global positioning system (GPS) coordinates of each site were recorded with a portable GPS (Fig.   1). All sites were generally remote with limited human presence; however, in Iceland and Norway where tourist activities are frequent, we aimed to sample at least 500 m away from human-related activities. At each location, three to five soil samples were collected within 100 m^2^ under the most common vegetation, for a total of 200 independent Arctic samples across 43 sites, with the aim to cover the broadest geographical area possible. Despite the extent, it should be noted that large areas of Canada and Russia remain uncharacterized and under-represented, highlighting areas where sampling should be prioritized in the future. Approximately 150 g of soil per sample was collected from the top 15 cm, avoiding plant roots and rocks and using an ethanol-cleaned shovel and Whirl-Pak bags (Nasco, WI, USA). Remaining plant roots and rocks were removed manually in a class II microbiological safety cabinet; samples were homogenized by manual mixing and frozen at −20°C before transportation to the United Kingdom. Samples were conserved at –20°C until analyzed.

### Soil properties

We focused our investigation on environmental variables previously identified as influencing bacterial communities and included pH, conductivity, moisture and organic carbon. Moisture content was measured gravimetrically for each soil sample after drying at 150°C for 24 h and total organic carbon (TOC) was measured gravimetrically by heating previously dried soils to 550°C for 4 h. pH and conductivity were measured in the laboratory in a 1:5 freshly thawed soil to water ratio, using a Mettler-Toledo FE20 pH meter (Mettler-Toledo Instruments Co., Shanghai, China) and a CMD500 conductivity meter (WPA, Cambridge, UK).

### DNA extraction

Soil DNA was extracted for each sample using the PowerSoil kit (Qiagen, Hilden, Germany), for a total of 400 DNA extracts. Each sample was amplified by polymerase chain reaction (PCR) using the universal primers 515F-806R, as per the Schloss lab standard operating procedure (Kozich *et al*. 2013) and the Earth Microbiome Project (Thompson *et al*. 2017), under the following conditions: initial denaturation at 95°C for 2 min then 30 cycles of 20 s denaturation at 95°C; primer annealing at 55°C for 15 s; elongation at 72°C for 5 min and then a final elongation at 72°C for 10 min. Negative controls, DNA extraction kit controls and ZymoBIOMICS mock communities (Zymo Research, Irvine, CA, USA) were included alongside the soil DNA and sequenced. PCR amplicons were cleaned and normalized using SequalPrep Plate Normalization Kit (Invitrogen, Carlsbad, CA, USA) and combined into four pools. The fragment size in each pool was assessed using the BioAnalyzer hsDNA assay (Agilent technologies, Santa Clara, CA, USA) and the concentration was measured using the Qubit hsDNA kit (Invitrogen). The library was supplemented with 5% PhiX and loaded on an Illumina MiSeq V2 500 cycles cartridge (Illumina, San Diego, Ca, USA).

### Illumina sequencing and data processing

Raw amplicon sequences were demultiplexed with the associated barcodes. Cutadapt (Martin 2011) was used for adaptor and primer clipping. Forward and reverse reads longer than 240 bp were merged (98% ± 0.8%/sample) using FLASH (fast length adjustment of short reads) (Magoč and Salzberg 2011) for a total of 20 million reads (∼50 000 ± 30 000 reads/sample) initially. Vsearch (Rognes *et al*. 2016) was used for downstream analyses. Quality filtering was carried out with an expected error <1.5. Dereplication was performed to identify unique sequences. A two-step chimera detection method was used, first by aligning against ChimeraSlayer Gold database provided with SILVA (Pruesse *et al*. 2007), second by using the *de novo* detection module in vsearch. *De novo* operational taxonomic unit (OTU) calling was performed on high-quality trimmed sequences at 97% similarity level using the USEARCH (Edgar 2010) algorithm for clustering implemented in vsearch to generate OTUs. Unique chimera filtered sequences were aligned using the Python Nearest Alignment Space Termination (PyNAST) (Caporaso *et al*. 2009) tool with a relaxed neighbor-joining tree built using FastTree (Price, Dehal and Arkin 2010). The taxonomy was determined using the Classification Resources for Environmental Sequence Tags (CREST) (Lanzén *et al*. 2012) classifier with a confidence threshold of 0.80 against SILVA release 128 as a reference database.

Samples less than at least 2000 reads/sample were filtered from the OTU table in order to have sufficient reads as described in Caporaso *et al*. (2011). After filtering, 386 samples were used for the statistical analyses, corresponding to 386 DNA extracts from 200 independent samples and ∼19.5 million reads (50 609 ± 26 700 reads/sample) assigned against 48 147 OTUs.

### Data availability

The dataset is deposited at European Nucleotide Archive/SRA under the accession number PRJEB29109.

### Statistical analysis

All statistical analyses were performed with a combination of QIIME1 V 1.90 (Caporaso *et al*. 2010) and the R environment (R Core Team 2013). Multiple rarefactions were performed in QIIME with the smallest sample size as maximum depth. Alpha diversity (richness, Shannon and Simpson indices) were calculated in QIIME on the matrices resulting from the multiple rarefactions. The differences in alpha diversity indices were tested in QIIME using nonparametric (Monte-Carlo) tests across different pH categories with a Bonferroni correction.

The nonrarefied OTU table was normalized using cumulative-sum scaling (CSS) in QIIME (Paulson *et al*. 2013). The resulting OTU table was input into R for subsequent analyses. Using the phyloseq package (McMurdie and Holmes 2013), we calculated the Bray–Curtis dissimilarity distance and visualized the ordination using principal coordinate analysis (PCoA). Using the cmdscale, envfit and ordisurf functions in the vegan package (Dixon 2003), we calculated and visualized the significant correlations between edaphic properties and bacterial community dissimilarity in relation to the pH gradient. Permutational multivariate analyses of variance (PerMANOVA) was conducted using the adonis function with 999 permutations. Pearson's correlation with 999 permutations was used to evaluate the collinearity of environmental variables.

To evaluate the spatial component, the geographic locations of the sampling sites were transformed into cartesian coordinates using the SoDA package (Chambers 2008) and the euclidean distance was calculated using the vegan package. The presence of a linear trend was tested by redundancy discriminant analysis and ANOVA as prescribed in Borcard, Gillet and Legendre (2018). A significant linear trend was identified, violating the second-order stationarity assumption where the mean of the variable and its spatial covariance are the same over the study area and its variance is finite. In other words, spatial correlation coefficients cannot be tested for significance if an overall trend is present in the data (Franklin and Mills 2007; Borcard, Gillet and Legendre 2018) and therefore, the data was detrended by linear regression of the *x*,*y* coordinates. To carry meaningful spatial analysis, constructing spatial variables representing spatial structures at all relevant scales is necessary (Borcard, Gillet and Legendre 2018). To do so, we used distance-based Moran's Eigenvector Maps (dbMEM) on detrended data and *x*,*y* coordinates using the adespatial R package (Dray *et al*. 2017). Significance of the spatial vectors was assessed using ANOVA. Forward selection was carried out to identify significant dbMEM vectors.

Variation partitioning analysis (VPA) was used to assess the impact of environmental and spatial factors on Bray–Curtis dissimilarity. The linear trend was considered to represent a source of variation like any other and therefore, we performed variation partitioning analysis using the environmental variables, *x*,*y* coordinates (trend) and significant dbMEM vectors.

Generalist and specialist taxa were identified by selecting taxa present in over 95% of samples within each unique and overlapped pH category. Indicator taxa were determined by the Dufrene-Legendre indicator species analysis using the multipatt function (Cáceres and Legendre 2009) to identify abundant OTUs that were specifically associated with the different pH ranges. The phylogenetic tree of indicator species was built using the aligned sequences from the identified indicator OTUs and input into FastTree. The tree was visualized using the Interactive Tree of Life (iTOL) online tool (Letunic and Bork 2016).

## RESULTS

### Bacterial community composition

We identified 48 147 bacterial taxa, mainly classified as Proteobacteria (20%) and Planctomycetes (15%). Acidobacteria represented 13% while Actinobacteria, Bacteroidetes, Chloroflexi and Verrucomicrobia each accounted for 4–6% of the community. Of these 48 147 OTUs, only 135 had overall abundances over 0.1% across all 386 samples and were defined as abundant taxa. They accounted for 32% of all the reads, illustrating the dominance of a few taxa over the rest of the community. Within these abundant taxa, Acidobacteria dominated the subcommunity at ∼32% with Blastocatellia (12.8%) and Acidobacteria subgroup 6 (7.2%) as abundant classes. Alphaproteobacteria (10.6%) and Betaproteobacteria (6.6%) were the most commonly identified Proteobacteria (21% overall) and Verrucomicrobia was largely represented by Spartobacteria (17.2%). Actinobacteria (10.7%), Chloroflexi (7%) and Bacteroidetes (3.5%) were also among the abundant phyla classified.

### Spatial and environmental factors influencing bacterial communities

**Figure 2.**
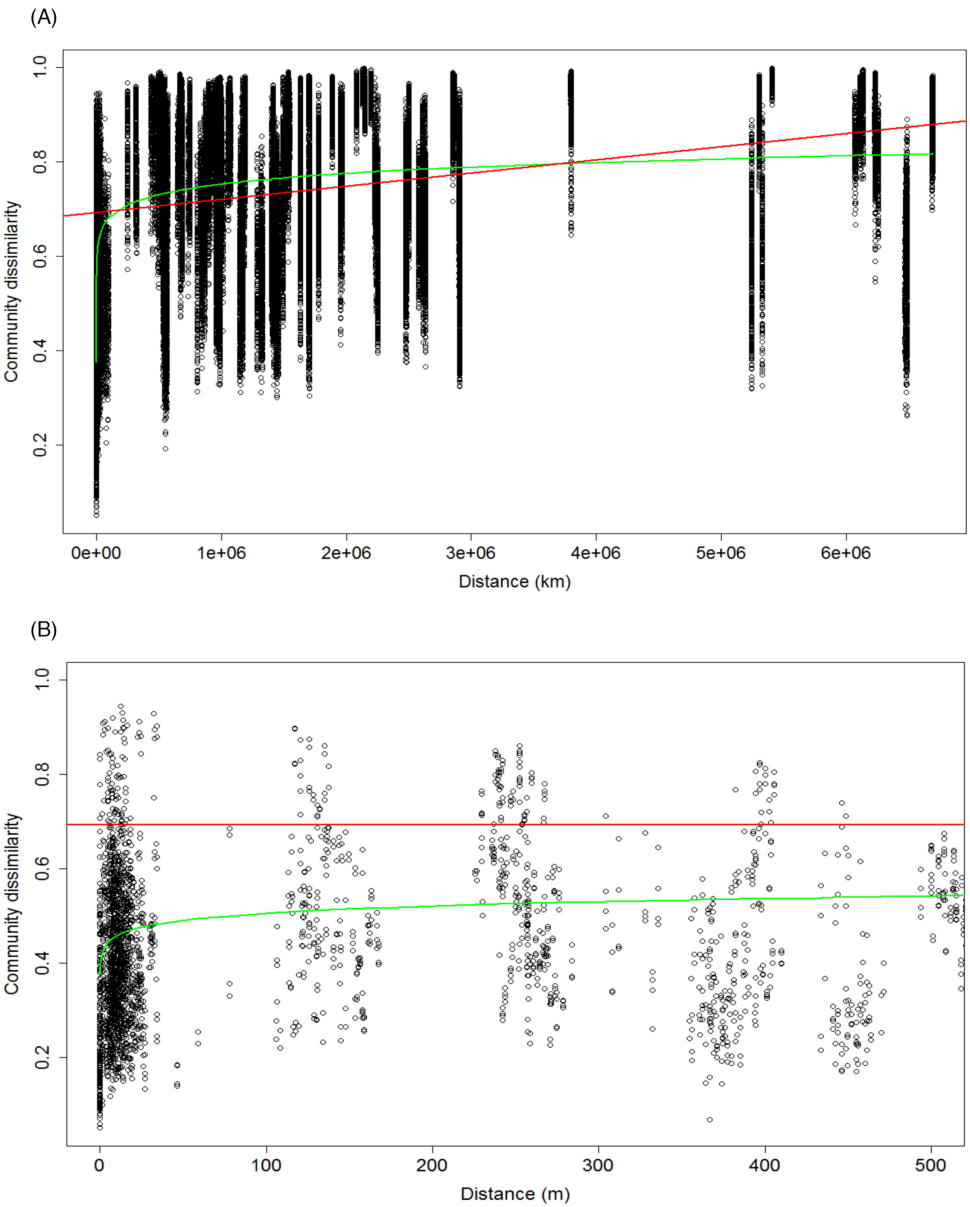
Distance–decay curves with the linear regression (red), the best-fitted power model (green). **(A)** Community dissimilarity of the total bacterial community by geographic distance in km. **(B)** Zoom into Fig. 2A from 0 to 500 m to assess the spatial autocorrelation distance.

Distance–decay curves were used to evaluate the influence of spatial factors on bacterial community structure. Figure 2A illustrates the increase in community dissimilarity with increasing geographic distance (linear regression: *R*^2^ = 0.108, *P <* 2.2 × 10*^−^*^16^). The best-fitted power model (*R*^2^ = 0.221, *P* = 0.005) suggested the presence of spatial autocorrelation, also qualified as dispersal limitation, within the first 500 m. For a more accurate estimate of dispersal limitation, we plotted the same graph within 0 and 500 m (Fig. 2B) and observed spatial autocorrelation approximately up to 20 m.

To determine the influence of environmental factors on the bacterial community structure, we used the adonis function with 999 permutations. Results indicated that pH explained 18.7% of the variance in bacterial community composition overall (Table 1). In comparison, conductivity, moisture and TOC explained almost 10% of the variance altogether. The measured environmental parameters explained 29% of the variance in the bacterial subcommunity, leaving 71% unexplained.

**Table 1.** The relative importance of environmental factors on bacterial communities, calculated by adonis.

	Total	
Variable	_*R*_2	Pr(*>*F)
pH	0.187	0.0001***
Conductivity	0.048	0.0001***
Moisture	0.027	0.0001***
TOC	0.021	0.0001***
Residuals	0.717	N/A

**Figure 3.**
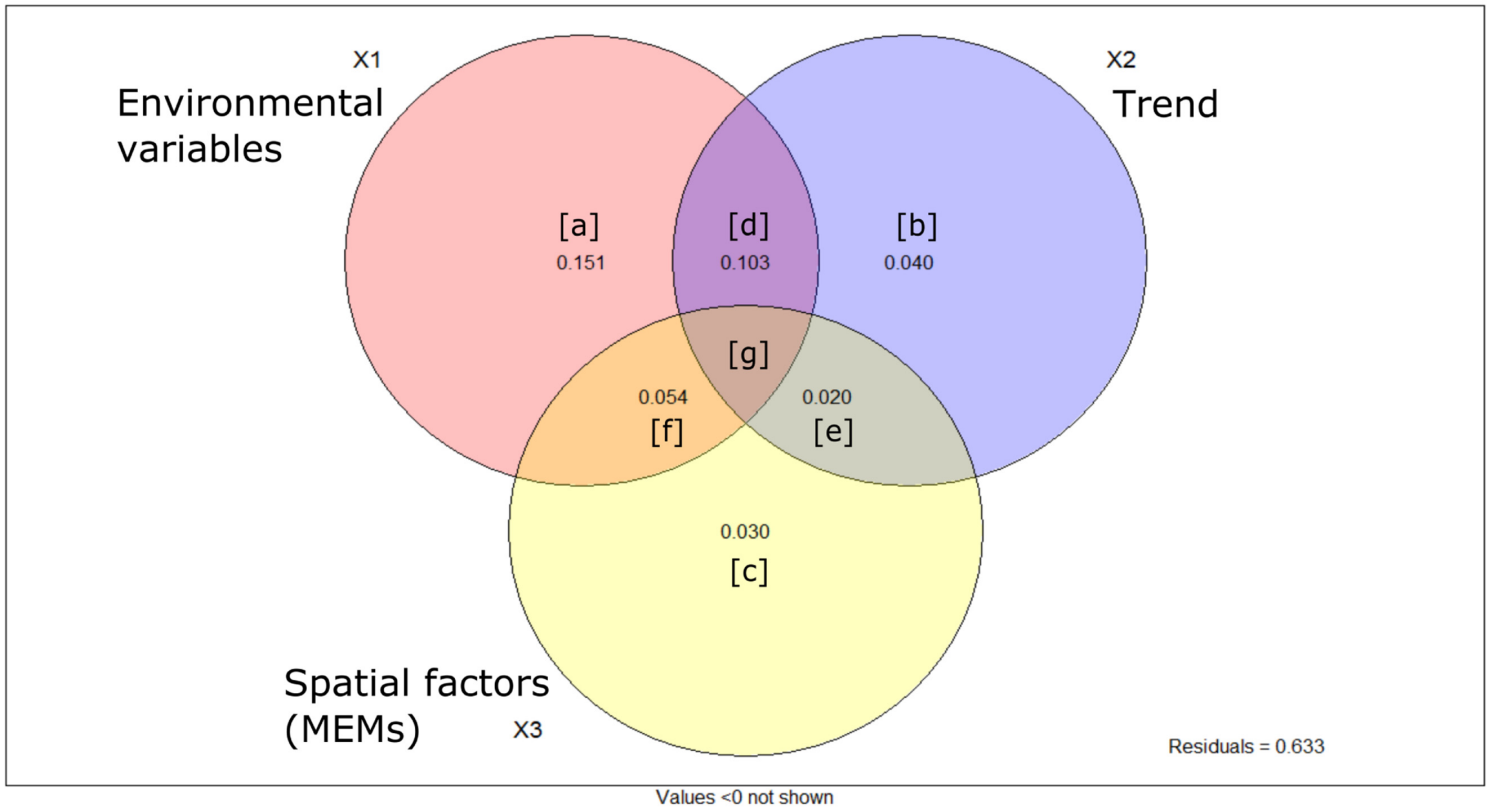
Venn diagram illustrating the results of the variation partitioning analysis and indicating the amount of variation in bacterial community explained by environmental variables and spatial factors including the linear trend. Detailed results are available in Table S1 (Supporting Information).

To quantify the influence of edaphic properties and spatial factors, we conducted variation partitioning on the bacterial community. We built spatial vectors using distance-based Moran's Eigenvector Maps (dbMEM) on *x*,*y* geographic coordinates. Three vectors (MEMs) were produced; after forward selection, two significant MEMs were identified for the total community and used in further analyses. The variation partitioning analysis differentiated the effect of environmental factors, linear trend and spatial vectors on Bray–Curtis dissimilarity (Fig. 3). Results indicated that the environmental factors (X1 = [a]+[d]+[g]+[f]) explained 30.7% of the variance in the community. These results are equivalent to the finding of the adonis test (Table 1), confirming the success of the variation partitioning analysis. Nonspatially structured environmental variables (fraction [a]) explained 15% of the variation while spatial factors only (fractions [b], [e] and [c]) explained ∼10% of the variation. Of the fraction X1 (environmental factors), 15% represented spatially structured environmental variables, also called induced spatial dependence, where the spatial structure of these environmental variables induced a similar spatial structure in the response data (detailed results in Table S1, Supporting Information). In total, 63.3% of the total bacterial community dissimilarity could not be explained by environmental and spatial factors after *R*^2^ adjustment.

### pH as a key environmental factor

**Figure 4.**
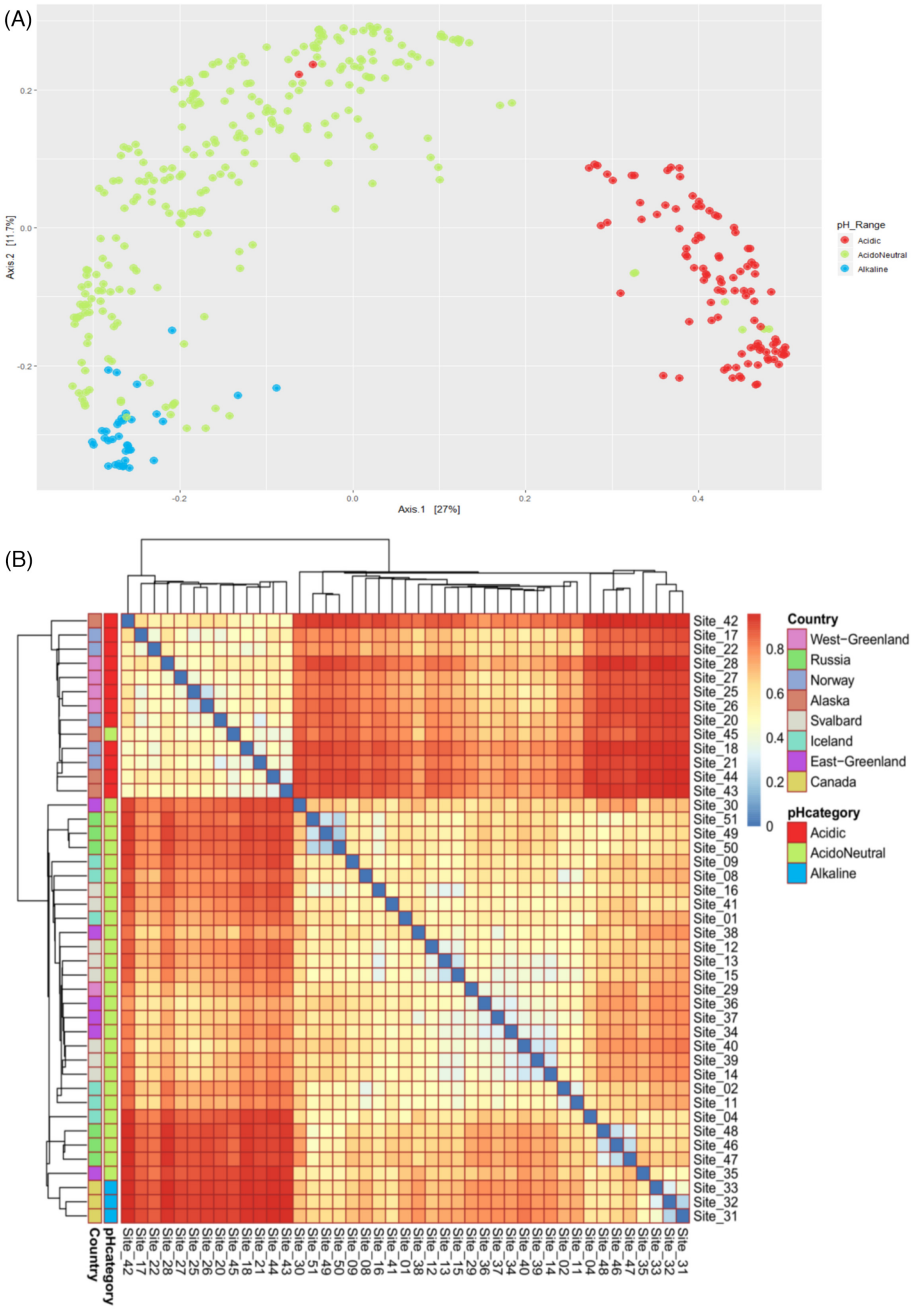
**(A)** PCoA of the Bray–Curtis dissimilarity of bacterial communities across the Arctic region by pH range. **(B)** Bray–Curtis dissimilarity heatmap and dendrogram illustrating the clustering of samples into three pH categories.

Of the environmental variables we measured, we identified pH as the primary factor structuring Arctic soil bacterial communities. The Pearson correlations indicated that pH did not have strong collinearity (coefficient over |0.8|) with any other variable measured (Table S2, Supporting Information). The clustering of samples by pH range was clearly observed on the PCoA of bacterial communities (Fig. 4A). The envfit analysis confirmed that pH was the key measured factor driving the ordination, although TOC, conductivity and moisture all had some influence. Using ordisurf, we visualized the PCoA ordination of the bacterial community in relation to the pH gradient (Figure S1, Supporting Information). The Bray–Curtis dissimilarity heatmap and dendrogram (Fig. 4B) identified three main clusters illustrating community differences. The first cluster was composed of acidic samples in a pH gradient from Norwegian soils at pH = 4.07 (±0.35) to samples from Alaska (pH = 4.64 ± 0.41) and West Greenland (pH = 4.90 ± 0.85). The second cluster included the lower acidoneutral range of samples from East Greenland (pH = 5.96 ± 0.69), Svalbard (pH = 5.65 ± 0.53) and Iceland (pH = 5.84 ± 0.46). Finally, the last cluster included the higher range of acidoneutral and alkaline soils from Russia (pH = 6.19 ± 0.22) and Canada (pH = 7.94 ± 0.67). The full spectrum of soil pH was covered from pH = 3.5 to pH = 9.0 and geographical locations often had samples from more than one pH category (Figure S2, Supporting Information). As pH was the primary factor we identified influencing bacterial community structure, we defined three pH categories to focus on: acidic, acidoneutral and alkaline.

### Bacterial diversity by pH category

**Figure 5.**
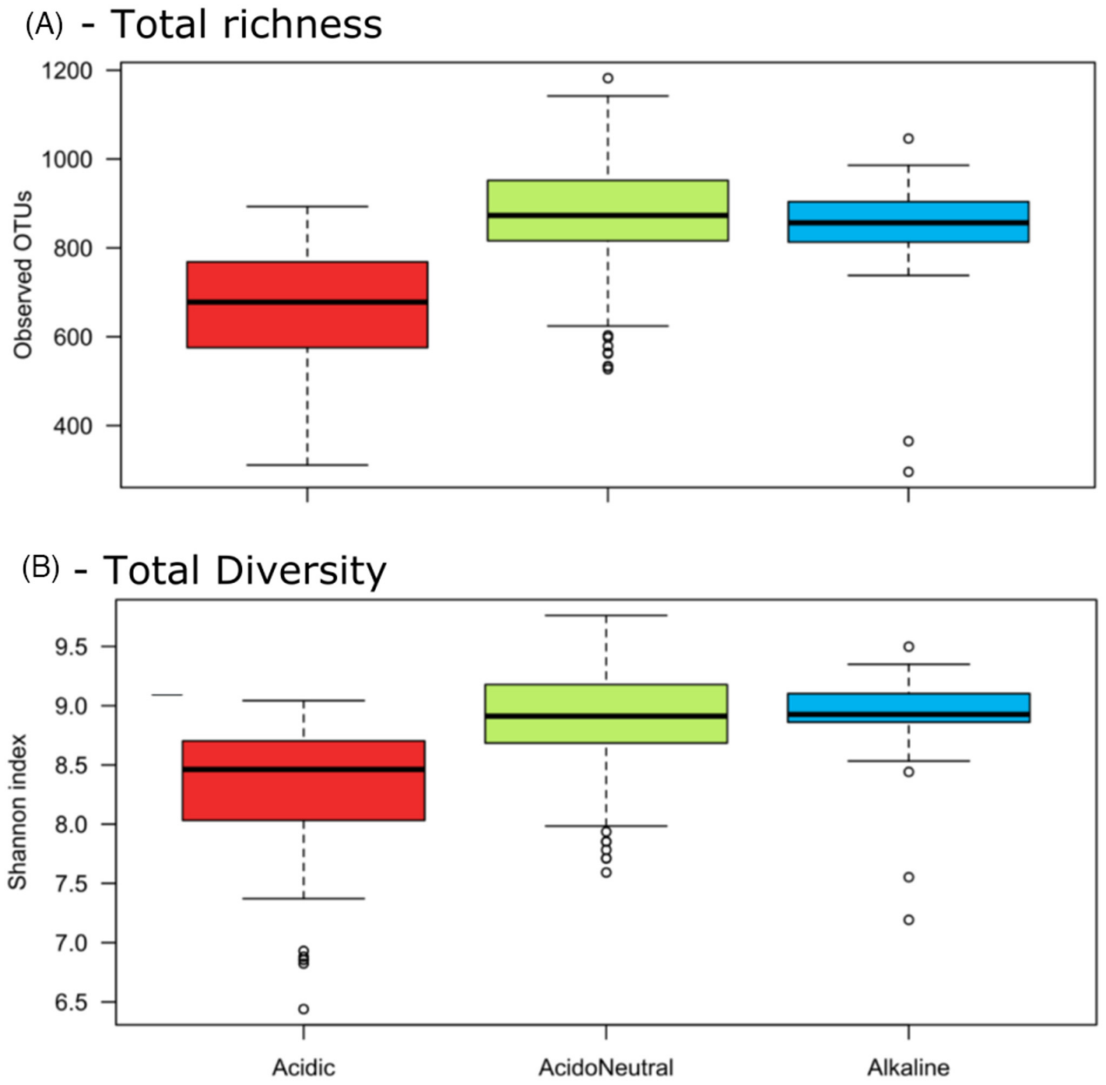
Alpha diversity differences in the total communities by pH category. **(A)** Richness by observed OTUs. **(B)** Shannon index.

We compared the observed richness and Shannon diversity index by pH range (Fig. 5A and B) using a Monte-Carlo test to assess differences. Alpha diversity was significantly lower in acidic samples than in acidoneutral and alkaline soils (Table S3, Supporting Information).

**Figure 6.**
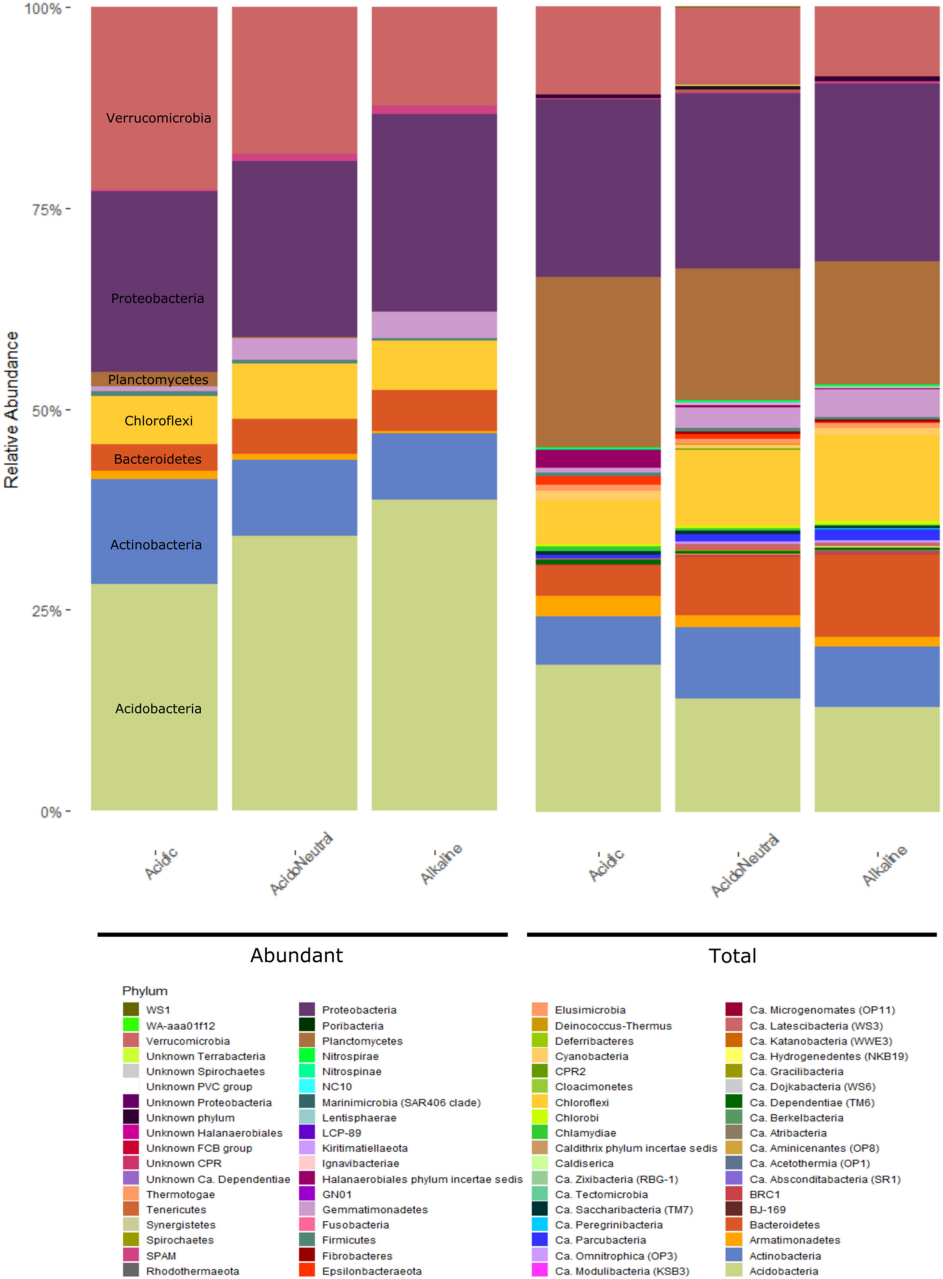
Relative abundance of the abundant and total communities by pH range classified at the phylum level.

In terms of community composition, we differentiated the abundant community, composed of only 135 OTUs, from the total community composed of 48 147 OTUs and observed the changes in relative abundance by pH range. In the total community, Acidobacteria, Armatimonadetes, Planctomycetes and Verrucomicrobia decreased with increasing pH. On the other hand, Bacteroidetes, Chloroflexi and Gemmatimonadetes increased in alkaline soils; Proteobacteria remained stable throughout. In the abundant community, Acidobacteria, Bacteroidetes and Gemmatimonadetes increased with increasing pH while Actinobacteria, Planctomycetes and Verrucomicrobia remained stable (Fig.   6).

### Generalist vs specialist taxa

**Figure 7.**
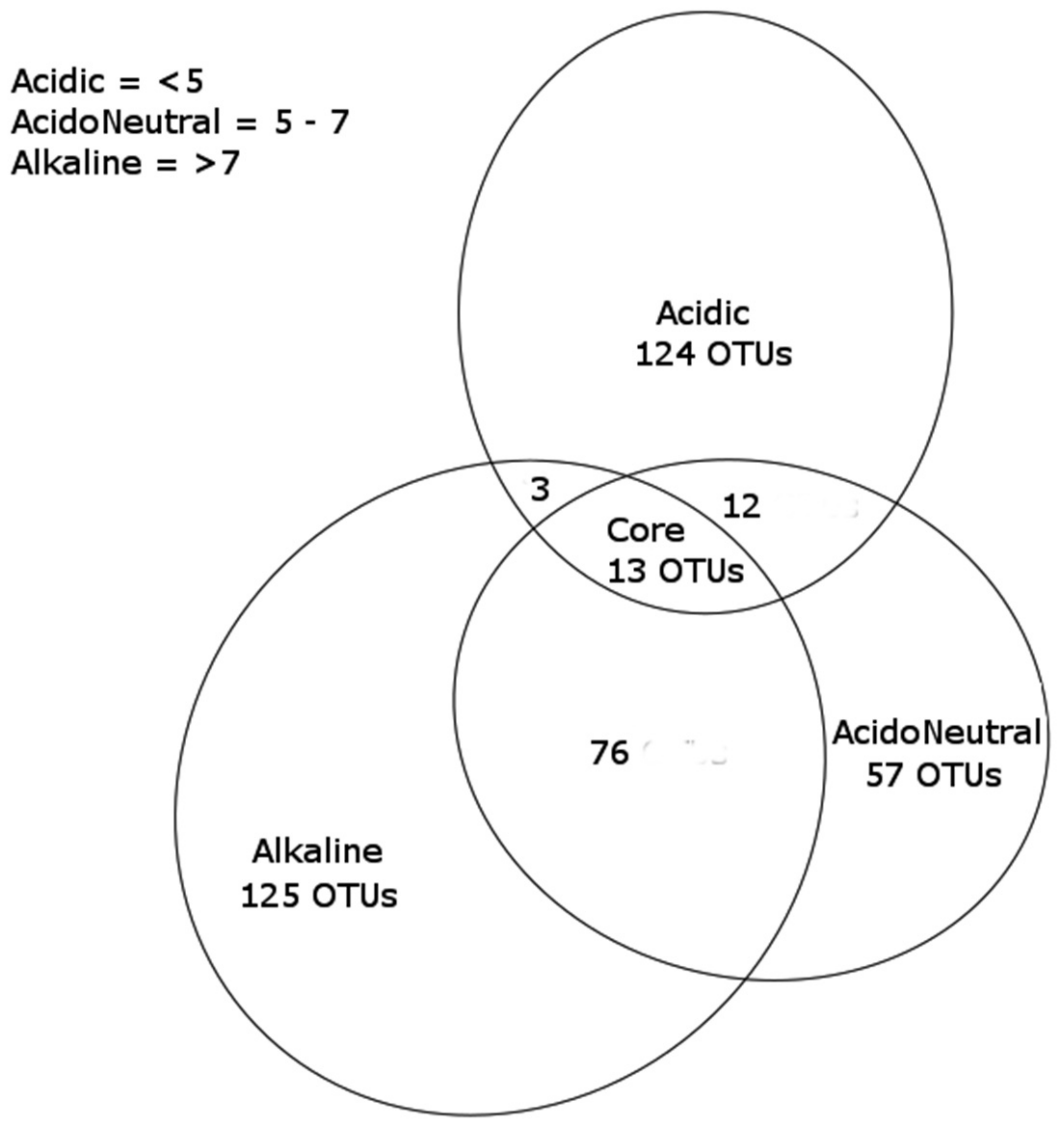
Venn diagram illustrating generalist (shared) and specialist (unique) OTUs by pH category.

The differentiation of specialist from generalist taxa associated with pH range was conducted by considering all taxa identified in this study and present in a minimum 95% of all samples from each pH category (Fig. 7). 125 acidic specialist OTUs were identified, unique to acidic soil samples. Of these 125 unique OTUs, most belonged to the Acidobacteria (27%), Verrucomicrobia (20%), Actinobacteria (14%), Planctomycetes (14%) and Proteobacteria (14%). At the class level, Acidobacteria group 1 dominated at 14%, while the rest of the identified classes had balanced relative abundances oscillating between 5 and 8%. Shared OTUs, or generalists were present in low numbers in acidic soils. 12 OTUs were shared with acidoneutral soils only, which were dominated by Alphaproteobacteria (65%), mainly Rhizobiales, while 3 OTUs were shared with alkaline only, belonging to the Spartobacteria (Verrucomicrobia), Phycisphaerae (Planctomycetes) and Chitinophagia (Bacteroidetes) classes. In comparison, acidoneutral soils had 76 shared taxa with alkaline soils, against 12 shared OTUs with acidic. Taxa shared with alkaline soils were mainly classified as Blastocatellia (Acidobacteria), Spartobacteria and Acidobacteria subgroup 6. Taxa exclusively found in acidoneutral soils belonged mainly to Actinobacteria (20%), Verrucomicrobia (20%) and Acidobacteria (19%). At the class level, unique taxa living in acidoneutral soils were dominated by Spartobacteria, Alphaproteobacteria and Holophagae. Alkaline soils presented a combination of both, a high number of shared (79 OTUs in total) and 125 exclusive taxa. Alkaline unique taxa were mostly composed of Acidobacteria (36%) and Proteobacteria (22%). From these unique taxa, the Acidobacteria subgroup 6 (20%), Blastocatellia (12%) and Alphaproteobacteria (12%) dominated the community.

### Indicator species

We conducted the indicator species analysis on the abundant taxa only as they may be more useful for monitoring purposes. We identified 17 unique taxa-habitat associations. In acidic soils, 10 indicator species, mainly Acidobacteria group 1 and 2 and Ca. Methylacidiphilum were identified. The six OTUs associated with Acidoneutral soils were mainly Acidobacteria group 4 (Blastocatellia) and Spartobacteria (Verrucomicrobia). Finally, only one OTU was identified as an indicator species for alkaline soils and belonged to the Holophagae (Acidobacteria). We also conducted the indicator species analysis of abundant taxa to combine pH ranges and identified 84 OTUs. 21 were identified as indicator species of acidic and acidoneutral soils combined, and mainly belonged to the Verrucomicrobia. Only two OTUs were associated with acidic and alkaline soils, Acidothermus (Actinobacteria) and Candidatus Xiphinematobacter (Verrucomicrobia), further illustrating the low overlap of taxa between these ecosystems and inferring the large ecosystem differences. Finally, 61 OTUs were associated with acidoneutral and alkaline soils, mainly belonging to the Acidobacteria and Proteobacteria phyla (see Table S3, Supporting Information for the detailed list). The phylogenetic tree (Figure S3, Supporting Information) illustrates the classification of the identified abundant indicator taxa with the pH range associated. Although displaying diversity, they were mainly classified as Acidobacteria and Verrucomicrobia, with the vast majority of taxa associated with acidoneutral and alkaline soils.

### The core microbiome

While some taxa displayed biogeographical patterns with nonrandom distribution across space and environmental gradients, others were cosmopolitan and identified in over 95% of all samples. The core microbiome represented 0.026% of bacterial communities and accounted for 2.7% of all reads. It was composed of 13 OTUs, mainly Proteobacteria and Acidobacteria, notably belonging to the Rhizobiales and Acidobacteria subgroup 6. The Alphaproteobacteria, especially the Bradyrhizobiaceae family, was most abundant in acidic soils and decreased with increasing pH. The Betaproteobacteria, especially the Comamonadaceae family, decreased with decreasing pH. The Acidobacteria were highest in the acidoneutral soils. Although relative abundances differed by pH range, these 13 OTUs were present in all categories. Figure S4 (Supporting Information) illustrates the taxonomic classes of the core microbiome and distribution between each pH category.

## DISCUSSION

The coverage of this study allowed the identification of a core microbiome, present in over 95% of the samples analyzed and composed of only 13 OTUs. The most abundant of these taxa belonged to the Bradyrhizobiaceae family. This is one of the most common families worldwide, as identified by Delgado-Baquerizo *et al*. (2018). However, whether this is the same taxa remains unclear and highlights the need for global studies incorporating extreme environments. The identification of a core Arctic soil microbiome is novel and this low number of cosmopolitan OTUs illustrates the potential for endemism. For the rest of the bacterial community, spatial and edaphic factors influenced distribution across the region.

### Arctic bacterial community assembly

The distance–decay curve (Fig. 2A and B) illustrated the influence of geography on bacterial communities. The spatial autocorrelation is a proxy for dispersal limitation, which was restricted to ∼20 m (Fig. 2B). However, the lack of small-scale data is problematic in estimating the real autocorrelation range. The variation partitioning analysis quantified the importance of both, selection (deterministic) and dispersal (stochastic), on the bacterial community structure. Environmental variables explained 30% of the total variation (selection), of which 15% was spatially structured, corresponding to the induced spatial dependence, as described in Borcard, Gillet and Legendre (2018). Spatial components (trend + MEMs) alone explained 9% of the variation, illustrating the spatial autocorrelation (Borcard, Gillet and Legendre 2018), or dispersal.

Here, we focused on environmental variables previously identified as influencing bacterial communities elsewhere and included pH, conductivity, moisture and organic carbon. However, 63% of the variation remained unexplained by either environmental or spatial factors in the variation partitioning analysis. This unexplained variance could be the result of unmeasured biotic or abiotic factors, directly or indirectly influencing bacterial communities. Examples of unmeasured edaphic properties could include total nitrogen, zinc, calcium oxide or nickel, all shown to have an influence on bacterial community structure in the Arctic (Wojcik *et al*. 2018). Other ecosystem properties such as ice presence, active layer depth or soil texture are likely to have some influence. Climatic and topographic variables such as temperature, precipitation (Yergeau and Kowalchuk 2008; Castro *et al*. 2010; Nielsen and Ball 2015) and altitude (Shen *et al*. 2013; Xu *et al*. 2014) are also known to influence bacterial assemblage and were not included in this study. Vegetation cover is a known driver of bacterial community assemblage in the Arctic region (as reviewed by Malard and Pearce (2018)) which was not included in this analysis. Biotic interactions may also be a major part of the unexplained variance observed and could include competition and predation within bacterial communities or with other members of the soil biota (Wardle 2006; Singh *et al*. 2009). Other processes influencing bacterial communities include diversification and drift, both likely to account for some of the unexplained variance (Martiny *et al*. 2006).

### The importance of pH

We evaluated the impact of environmental variables known to influence global soil bacterial communities. Overall, pH explained the most variation (18.7%) in bacterial community structure and diversity while TOC, moisture and conductivity explained between 2 and 5% of the variance, respectively. The identification of pH as a key factor influencing Arctic soil bacterial community composition is in line with previous Arctic studies over both, small and large scales (Männistö, Tiirola and Häggblom 2006; Chu *et al*. 2010; Ganzert, Bajerski and Wagner 2014; Siciliano *et al*. 2014; Schostag *et al*. 2015). However, this study is the first to quantify the magnitude of influence, highlighting the large variation in bacterial community remaining unexplained. While pH has been identified globally as a major factor influencing microbial diversity and community structure (Fierer and Jackson 2006; Lauber *et al*. 2009; Tedersoo *et al*. 2014; Delgado-Baquerizo *et al*. 2018), the underlying processes and mechanisms by which it does remain unclear. Studies have demonstrated that the soil pH is correlated with other elements of the geochemistry and has a strong impact on nutrient and water availability as well as solubility and adsorption (Gray *et al*. 2014). For instance, acidic pH increases aluminum, hydrogen and manganese solubility, retarding plant root growth due to high toxicity (Clark and Baligar 2000; Singh *et al*. 2017). Acidic soils have not only nutrient deficiencies such as calcium magnesium and potassium but also decreased phosphorus and molybdenum solubilities (Clark and Baligar 2000; Gray *et al*. 2014). Alkaline soils are generally the result of low precipitation and high evapotranspiration, leading to low water availability and in common with acidic soils, nutrient deficiencies are found with decreased phosphorus, iron, copper or zinc for instance (Clark and Baligar 2000). In similar ways, acidic and alkaline soils are generally considered harsh environments requiring a wide range of adaptations from microorganisms while acidoneutral soils are considered the optimum environment for microbial life (Fierer and Jackson 2006; Rousk *et al*. 2010); such differences in soil composition are likely driving the observed differences in microbial community composition by pH range.

### Distribution of generalist and specialist taxa

Microbial communities are assembled by deterministic (selection) and stochastic (dispersal) processes. It has been hypothesized that communities primarily structured by deterministic processes will host more specialist taxa, highly adapted to the ecosystem, while communities influenced by dispersal will harbor primarily generalist taxa, more resilient to change (Pandit, Kolasa and Cottenie 2009; Graham and Stegen 2017; Sriswasdi, Yang and Iwasaki 2017). While specialist taxa are restricted to certain niches, they can be locally abundant; shared taxa, or generalists, are distributed across many niches (Barberán *et al*. 2012). In most cases, specialist will be more abundant because generalists rapidly become specialists to adapt to their ecosystems, despite generalists having evolutionary advantages (Sriswasdi, Yang and Iwasaki 2017).

In this study, bacterial communities were specialist-dominated in acidic soils, generalist-dominated in acidoneutral soils and a mixed community in alkaline samples. The higher abundance of specialists in acidic soils (considered the harshest systems) could illustrate the need for environmental adaptations to survive in these ecosystems and suggests that deterministic processes likely structure microbial communities. Geographically, the first cluster identified by the Bray–Curtis dissimilarity matrix (Fig. 4B) grouped all acidic samples from northern Norway, western Greenland and Alaska, illustrating the similarities of their bacterial communities despite the large distances separating these locations, further suggesting the strong influence of selection over dispersal. The dominance of generalists in acidoneutral soils illustrates the lower environmental pressure to have specific survival adaptations and infers the dominance of stochastic processes in community structuring. Eastern Greenland, Svalbard and Iceland were grouped together by the Bray–Curtis dissimilarity matrix. Geographically, these locations connected through the Greenland sea, a possible route of dispersal through aerosolization and long-distance aeolian transport (Šantl-Temkiv *et al*. 2018). Finally, alkaline soils hosted a mixed community of specialists and generalists, suggesting the combination of both, selection and dispersal, in community structuring. The abundance of generalist taxa in alkaline soils, generally considered a harsh system in many ways similar to acidic soils, highlights the adaptability of generalists to a wide range of environmental conditions. Interestingly, the Bray–Curtis dissimilarity matrix clustered the Canadian (alkaline) and Russian (acidoneutral) samples together. The Russian samples were on the higher end of the acidoneutral pH scale and the grouping of these samples illustrates the blurred boundary between acidoneutral and alkaline pH categories. While environmental selection may be driven by many variables, dispersal likely occurs via long-distance aeolian dispersal (Šantl-Temkiv *et al*. 2018).

### The importance of indicator taxa

The indicator species analysis determined OTU-pH range associations identifying different classes of Acidobacteria and Verrucomicrobia, primarily, characteristic of each pH range. The identification of abundant indicator species with strong habitat associations opens the possibility of predicting the presence and abundance of these taxa across the Arctic region. This is especially important for taxa such as Ca. Methylacidiphilum, a known methanotroph unlike others as it belongs to the Verrucomicrobia phylum instead of the Proteobacteria (Dunfield *et al*. 2007; Khadem *et al*. 2010). While natural CH_4_ emissions come primarily from wetlands, the identification of these phylotypes in acidic soils suggests wetland may not be the only major source of northern methane emissions. Understanding the distribution and abundance of such indicator taxa, combined with field gas measurements, may allow large-scale estimates of climate-active gases release rates in the region, from soils and wetlands (Wartiainen *et al*. 2006; Jørgensen *et al*. 2015).

## CONCLUDING REMARKS

This study investigated patterns in soil bacterial diversity across the Arctic and demonstrated that bacterial communities differed across the region. However, the dispersal range was limited to ∼20 m and the variation in communities across the Arctic was primarily driven by environmental rather than spatial factors. The key edaphic property influencing communities we identified was pH, in accordance with that seen elsewhere. However, 63% of the variation remained unexplained, calling for more in-depth investigations of the drivers of bacterial community structure in the region. We identified unique and indicator taxa closely associated to the soil pH. Finally, a core microbiome composed of only 13 ubiquitously distributed taxa was identified, highlighting the potential for endemism in the region. While this study brings a deeper understanding of Arctic bacterial community assemblages, this is also a baseline for future functional studies in the region, which will be critical to forecast the ecological consequences of environmental change.

## Supplementary Material

fiz128_Supplemental_Files

## Author Biographical

LAM and DAP conceived and designed the study and sampling strategy. LAM carried the fieldwork and laboratory work. MZA conducted bioinformatics processing, LAM and MZA conducted the statistical analysis. LAM drafted the manuscript and MZA, DAP and CSJ revised and approved the final version.
