# Supplementary figures and images for: Biogeographical patterns in soil bacterial communities across the Arctic region
Source: FEMS Microbiol Ecol. 2019 Aug 20;95(9):fiz128. doi: 10.1093/femsec/fiz128 (PMC6736398; doi:10.1093/femsec/fiz128)

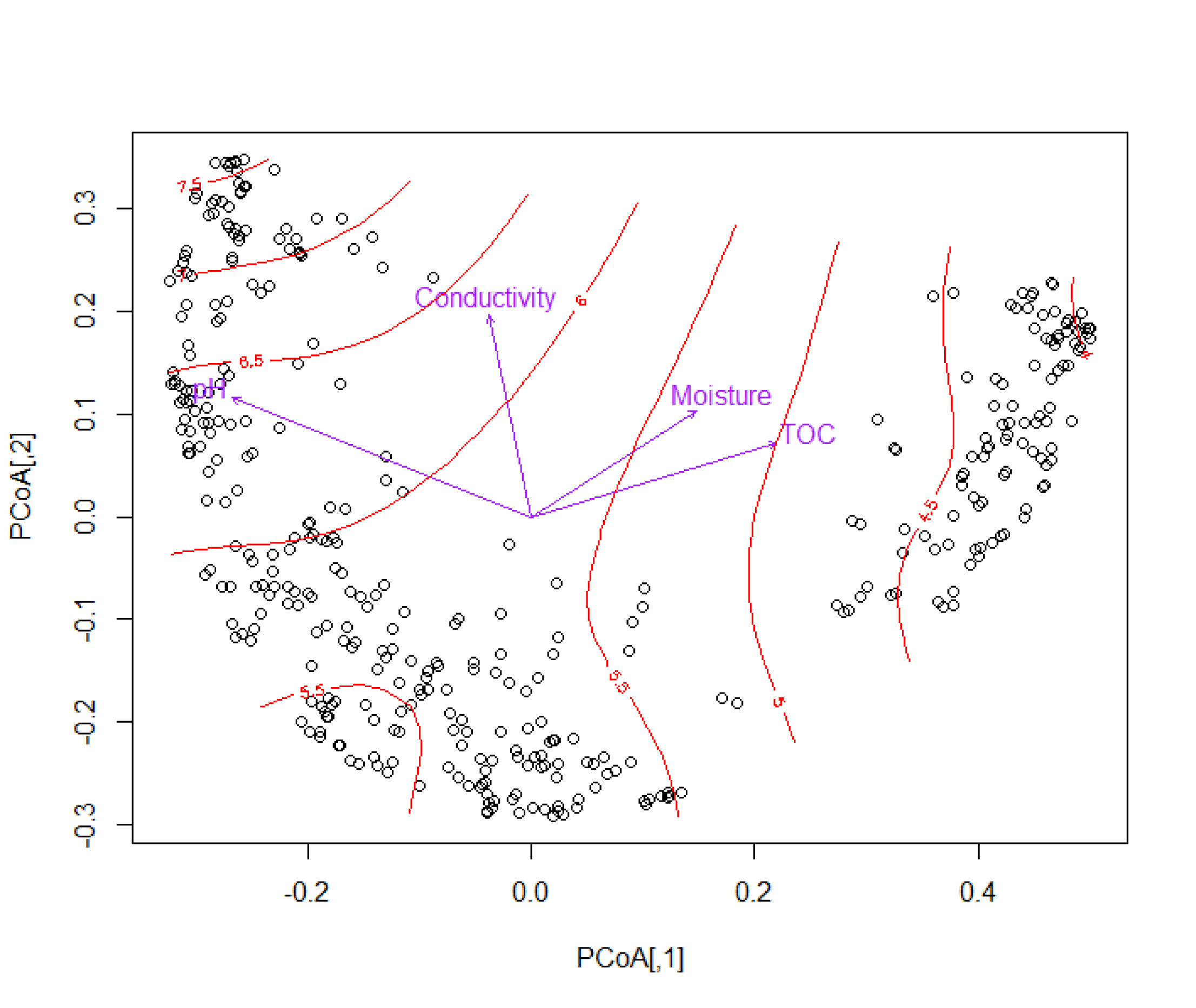

Supplement: fiz128_Supplemental_Files [file fiz128_supplemental_files.zip › Fig.S1.tif]

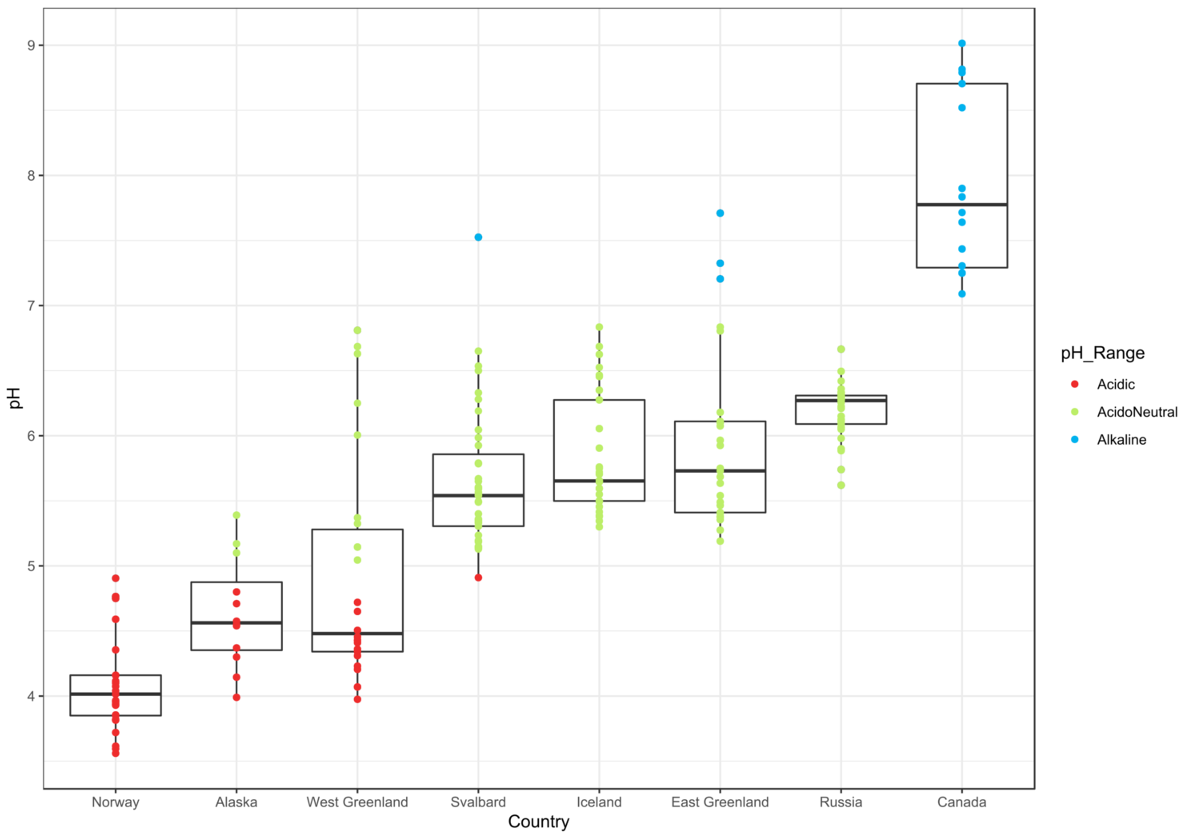

Supplement: fiz128_Supplemental_Files [file fiz128_supplemental_files.zip › Fig.S2.tif]

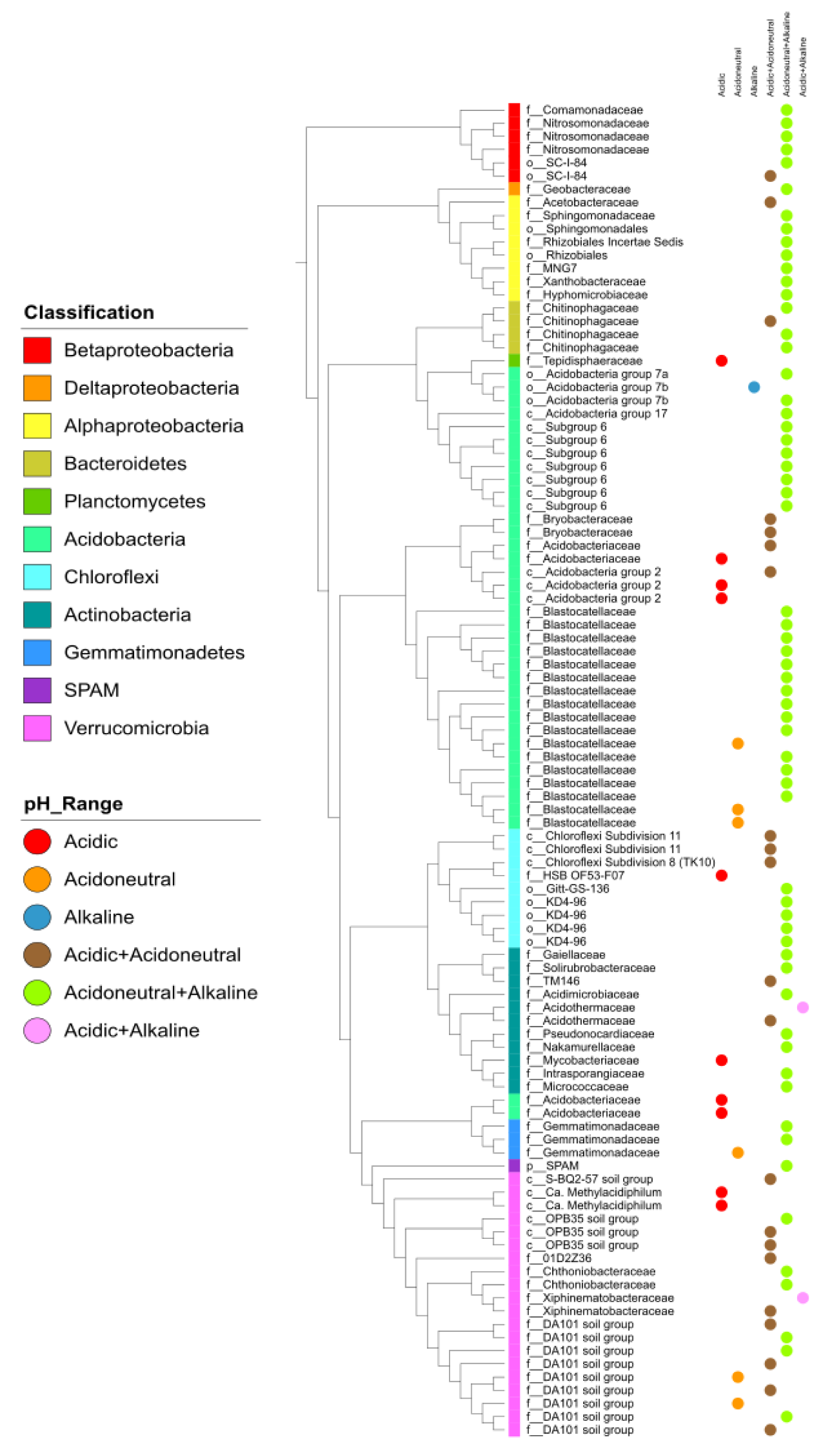

Supplement: fiz128_Supplemental_Files [file fiz128_supplemental_files.zip › Fig.S3.tif]

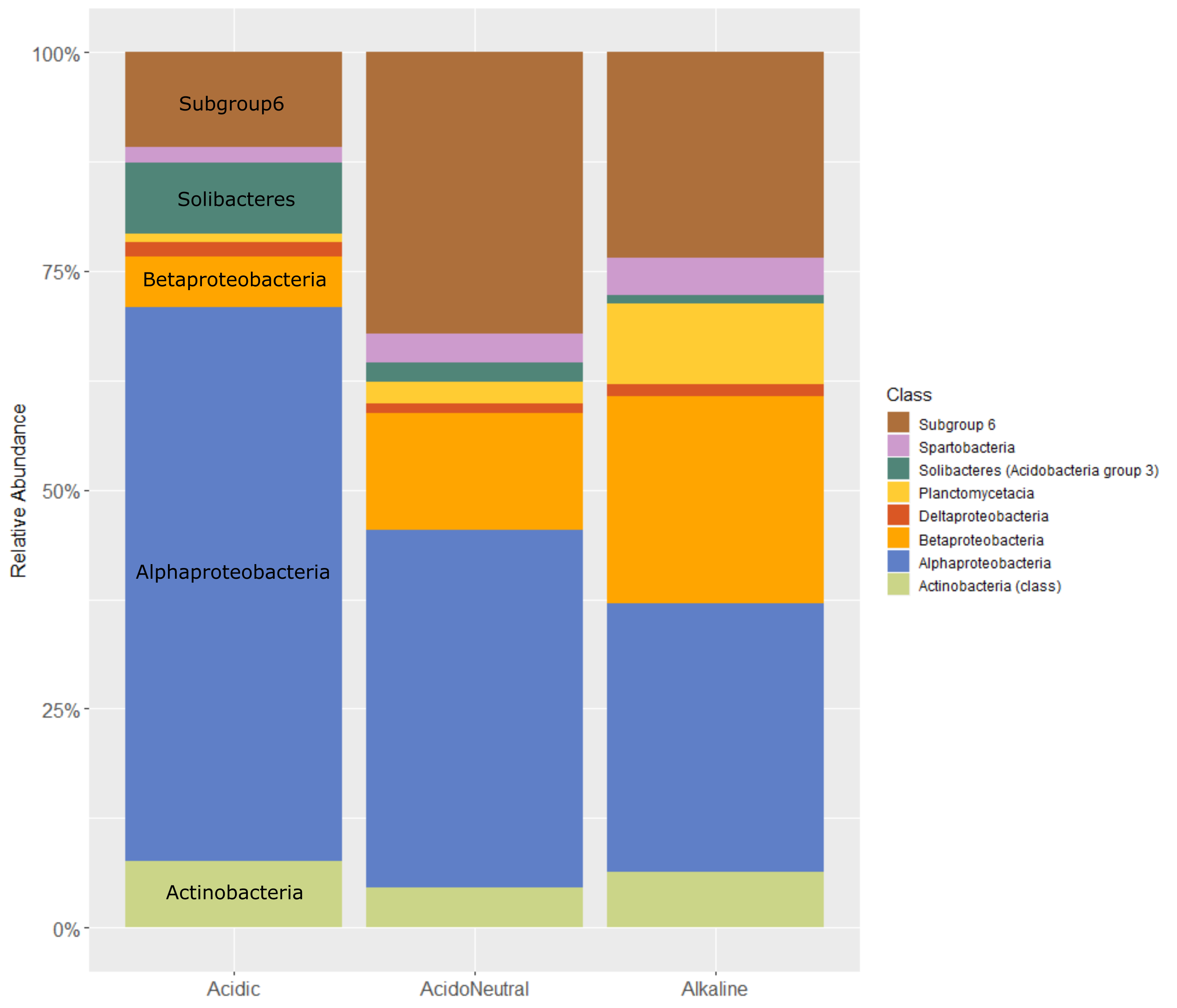

Supplement: fiz128_Supplemental_Files [file fiz128_supplemental_files.zip › Fig.S4.tif]
